# Supplementary material for: Causal Relationship between Aging and Anorexia Nervosa: A White-Matter-Microstructure-Mediated Mendelian Randomization Analysis
Source: Biomedicines. 2024 Aug 16;12(8):1874. doi: 10.3390/biomedicines12081874 (PMC11351342; doi:10.3390/biomedicines12081874)
Supplement: Supplementary file 1 [file biomedicines-12-01874-s001.zip › Supplementary Figure/Figure S4/Singe SNP plot of MR analysis for TL to ubm-b-1738.pdf]

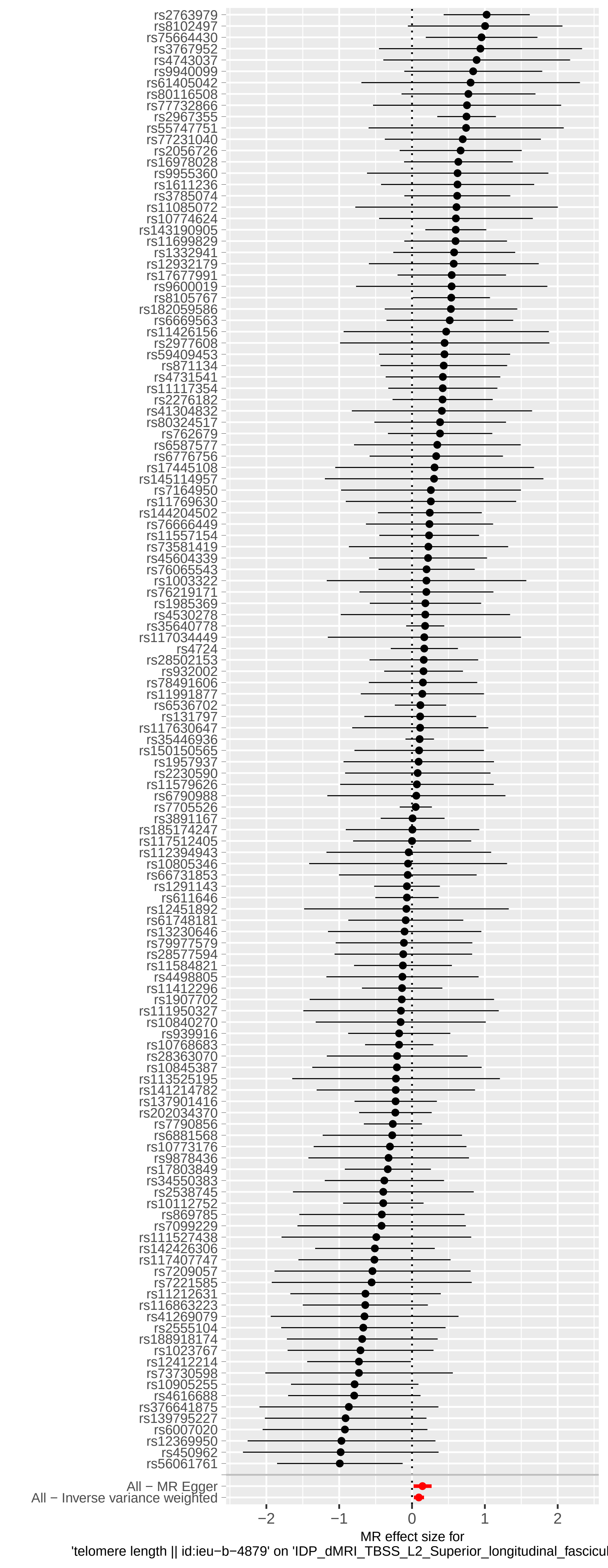
rs2763979
rs8102497
rs75664430
rs3767952
rs4743037
rs9940099
rs61405042
rs80116508
rs77732866
rs2967355
rs55747751
rs77231040
rs2056726
rs16978028
rs9955360
rs1611236
rs3785074
rs11085072
rs10774624
rs143190905
rs11699829
rs1332941
rs12932179
rs17677991
rs9600019
rs8105767
rs182059586
rs6669563
rs11426156
rs2977608
rs59409453
rs871134
rs4731541
rs11117354
rs2276182
rs41304832
rs80324517
rs762679
rs6587577
rs6776756
rs17445108
rs145114957
rs7164950
rs11769630
rs144204502
rs76666449
rs11557154
rs73581419
rs45604339
rs76065543
rs1003322
rs76219171
rs1985369
rs4530278
rs35640778
rs117034449
rs4724
rs28502153
rs932002
rs78491606
rs11991877
rs6536702
rs131797
rs117630647
rs35446936
rs150150565
rs1957937
rs2230590
rs11579626
rs6790988
rs7705526
rs3891167
rs185174247
rs117512405
rs112394943
rs10805346
rs66731853
rs1291143
rs611646
rs12451892
rs61748181
rs13230646
rs79977579
rs28577594
rs11584821
rs4498805
rs11412296
rs1907702
rs111950327
rs10840270
rs939916
rs10768683
rs28363070
rs10845387
rs113525195
rs141214782
rs137901416
rs202034370
rs7790856
rs6881568
rs10773176
rs9878436
rs17803849
rs34550383
rs2538745
rs10112752
rs869785
rs7099229
rs111527438
rs142426306
rs117407747
rs7209057
rs7221585
rs11212631
rs116863223
rs41269079
rs2555104
rs188918174
rs1023767
rs12412214
rs73730598
rs10905255
rs4616688
rs376641875
rs139795227
rs6007020
rs12369950
rs450962
rs56061761
All − MR Egger
All − Inverse variance weighted
−2
−1
0
1
2
MR effect size for
'telomere length || id:ieu−b−4879' on 'IDP_dMRI_TBSS_L2_Superior_longitudinal_fascicul
